# Measures of Implicit and Explicit Adaptation Do Not Linearly Add

**DOI:** 10.1523/ENEURO.0021-23.2024

**Published:** 2024-08-23

**Authors:** Bernard Marius ‘t Hart, Urooj Taqvi, Raphael Q. Gastrock, Jennifer E. Ruttle, Shanaathanan Modchalingam, Denise Y. P. Henriques

**Affiliations:** ^1^York University, Toronto, Ontario M3J 1P3, Canada; ^2^University of Waterloo, Waterloo, Ontario N2L 3G1, Canada

**Keywords:** adaptation, additivity, explicit, implicit, reaching

## Abstract

Moving effectively is essential for any animal. Thus, many different kinds of brain processes likely contribute to learning and adapting movement. How these contributions are combined is unknown. Nevertheless, the field of motor adaptation has been working under the assumption that measures of explicit and implicit motor adaptation can simply be added in total adaptation. While this has been tested, we show that these tests were insufficient. We put this additivity assumption to the test in various ways and find that measures of implicit and explicit adaptation are not additive. This means that future studies should measure both implicit and explicit adaptation directly. It also challenges us to disentangle how various motor adaptation processes do combine when producing movements and may have implications for our understanding of other kinds of learning as well (data and code: https://osf.io/3yhw5).

## Graphical abstract

**Figure:**
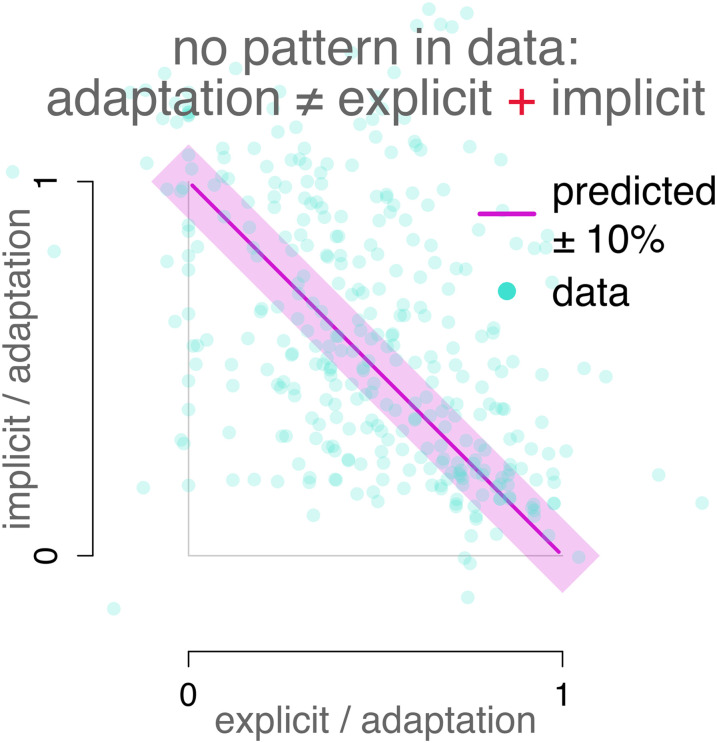
Visual Abstract

## Significance Statement

Multiple processes contribute when we adapt our movements to changing circumstances. Currently, these are often grouped into implicit and explicit processes. How these are combined in actual behavior has not been well studied. Nevertheless, it seems common practice in the field to assume that measures of implicit and explicit adaptation can simply be added to get a valid estimate of total adaptation. Here we test this assumption and find that it can not be relied on. This means that for now, we need independent measures of each process. Further, we should examine the neural mechanisms by which independent learning processes may each contribute to overt behavior.

## Introduction

Both implicit (unconscious, automatic) and explicit (conscious, intentional) processes contribute to various kinds of learning (Jacoby, 1991). Research exploring the contribution of implicit and explicit processes to human motor adaptation relies on the notion that these processes are related (Benson et al., 2011; Taylor and Ivry, 2011; Werner et al., 2015). Many recent studies assume they linearly add to total adaptation, with some support (Redding and Wallace, 1993; Sülzenbrück and Heuer, 2009; Bond and Taylor, 2015). Here, we test whether this additivity assumption holds.

The main idea is that there are only two kinds of processes contributing to adaptation: those we are aware of and those we are not aware of. The processes we are aware of, which are usually intentional, involve a strategy, can be verbalized, and are often effortful, are referred to as “explicit” adaptation. The processes we are not aware of are then not intentional but automatic, they can not be verbalized and since they are automatic they usually require less effort, and these kinds of processes are often called “implicit” adaptation. A further distinction is that explicit processes can be voluntarily disengaged, as opposed to implicit processes. Implicit and explicit adaptation could each be split up further or overlap with other kinds of processes (e.g., reward-based learning could be both implicit and explicit). However, if we can split motor adaptation into implicit and explicit processes, it seems intuitive that we can also add them for an estimate of total adaptation, the “additivity assumption” (Maresch et al., 2021a):

adaptation∼=implicit+explicit.

**Figure 1.**
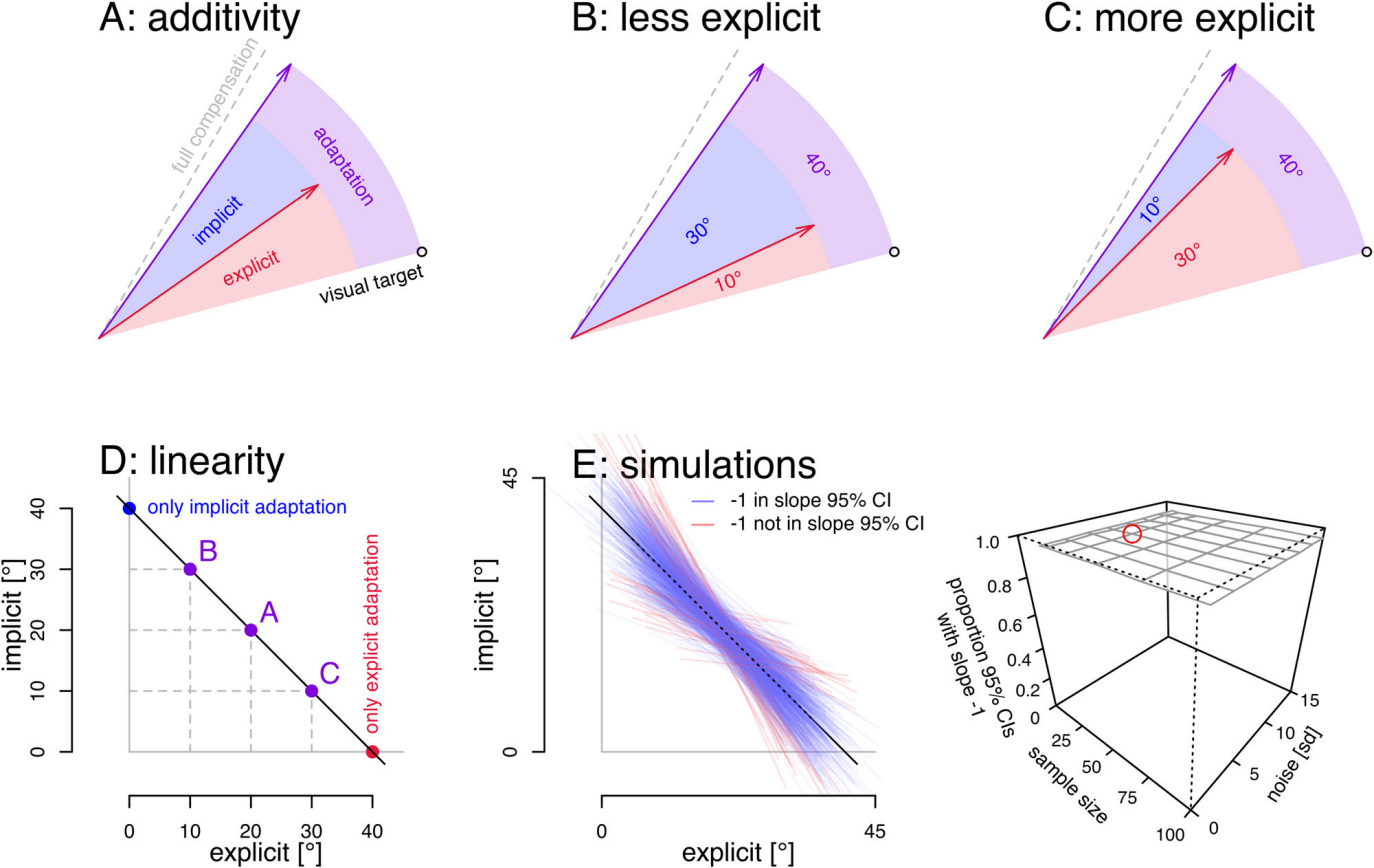
The additivity assumption. ***A***, Often, implicit adaptation (blue) is considered what is left after explicit adaptation (red) is subtracted from total adaptation (purple). ***B***, ***C***, Given less explicit adaptation, this would mean there is more implicit adaptation (***B***) and vice versa (***C***). ***D***, This means that if explicit and implicit adaptation are additive, they should have a negative, linear relationship. The purple points depict the situations in panels ***A–C*** and also shown are situations where adaptation is fully implicit (blue) and fully explicit (red). ***E***, We generated 1,000 data sets (sample size, 24) with the mechanism prescribed by the additivity assumption adding normally distributed noise (SD: 7) and test if we can recover the predicted negative slope from a linear model fitted to each data set (left). In ∼95% of simulated data sets, the 95% confidence interval of the slope includes the predicted value of −1 (blue lines) and in ∼5% it does not (red lines). Finally, we test different sample sizes and levels of noise but that does not affect the results of our test: in all cases of ∼95% of the simulated data sets, the 95% CI for the slope includes −1 (right). Red circle, the combination of sample size and noise used on the left.

Often illustrated as in Figure 1*A*, this relationship, if true, could be rewritten as follows:

implicit∼=adaptation−explicit.

In Figure 1*A*, implicit adaptation (blue wedge) is depicted as what is left when explicit adaptation (red arrow) is subtracted from total adaptation (purple arrow). In theory, the additivity assumption allows researchers to determine the implicit contributions to motor adaptation without the need to directly measure implicit learning. In practice however, the underlying assumption is not always replicated (Modchalingam et al., 2019) and has been called the “least robust” assumption in motor adaptation (Maresch et al., 2021a).

Some of the papers our lab published in the last 5 years (Modchalingam et al., 2019; Gastrock et al., 2020; Vachon et al., 2020) used the Process Dissociation Procedure (PDP; Jacoby, 1991; Werner et al., 2015) to assess implicit as well as explicit adaptation. In the first paper we already noticed that reach deviations in include strategy no-cursor trials did not equal the reach deviations during training trials. That is, the difference between include and exclude no-cursor reach deviations would not necessarily equal the magnitude of explicit strategies. Hence, in the two later papers, we just took the existence of any difference as definite evidence for an explicit strategy. This also leads us to question the additivity of explicit and implicit adaptation. In particular, it seemed to us that there are two very separate neural processes that likely manifest in different brain areas or even networks. It would be very unlikely for such different processes to linearly add—which would simply not be a biologically plausible mechanism. It may also have to do with the nature of the measurements used in the PDP, but these do have one distinct advantage: there are two no-cursor measures as well as adaptation. Using these three different measures, it is possible to notice any kind of discrepancy resulting from nonadditivity. Nevertheless, we should not have relied on additivity and our PDP-style approach to assess explicit adaptation.

In recent motor learning work, explicit adaptation is instead measured as reaiming reports. While this provides a better measure of explicit adaptation, it is often combined with the additivity assumption to forego measuring implicit adaptation. Instead, implicit adaptation is taken as the difference between total adaptation and explicit adaptation. A substantial portion of findings rely on such “indirect” means to determine implicit adaptation (McDougle et al., 2015; Miyamoto et al., 2020; Wilterson and Taylor, 2021).

Both the PDP-style approach and reaiming-based approaches on their own rely heavily on the additivity assumption as they either have no direct measure of explicit adaptation or no direct measure of implicit adaptation. In a set of experiments we attempt to validate if the assumption of additivity would allow for the “indirect” method of determining implicit or explicit learning to be equivalent to direct measures. In particular, the field seems to focus on using reaiming measures and subtracting them from total adaptation as a measure of implicit learning. So this is where we will focus as well, although for completeness we also test additivity in data collected with a PDP approach.

The statistical approach we follow relies on the negative correlation between implicit and explicit adaptation that would be dictated by additivity. That is, when total adaptation is constant, and explicit adaptation decreases, implicit adaptation has to increase by the same amount (Fig. 1*B*) and vice versa, when explicit adaptation increases, implicit adaptation decreases (Fig. 1*C*). That relationship between implicit and explicit adaptation under additivity can be plotted as a linear function with an intercept equal to total adaptation and a slope of −1 (Fig. 1*D*). To see if this would be reproducible in principle, we ran a simulation where we added noise to data generated with an additive model. All noise (ϵ) was drawn separately from a normal distribution with $\mu =0^{\circ }$ and $\sigma =7^{\circ }$ (results are independent of the level of noise). For each participant (p), total adaptation and explicit adaptation are given by the following:

$$A_{p}=40^{\circ }+\epsilon_{a,p},$$

$$E_{p}=20^{\circ }+\epsilon_{e,p}.$$

And implicit adaptation is taken as the difference between total adaptation and explicit adaptation (plus noise):

$$I_{p}=A_{p}+E_{p}+\epsilon_{i,p}.$$

We can repeat this for a number of participants and then fit a linear model predicting the generated implicit adaptation from the corresponding explicit adaptation. We will see how well a linear model fitted to the generated data recovers the slope of −1. (The intercept should be close to total adaptation, but since most of the data should be somewhat removed from zero explicit adaptation, the intercepts will vary much more and this might not be as good a test.) In particular we can test if the 95% confidence interval of the mean for the slope parameter includes −1. Using samples of 24 participants (equal to our experiment, see below) we have repeated this 1,000 times (linear fits shown in Fig. 1*E*, left), and in ∼95% of simulations the 95% confidence interval of the slope includes −1 (blue lines in Fig. 1*E*; red lines: 95% CI for the slope excludes −1). Finally, with 10,000 simulations each for various combinations of sample size and noise, we show that this statistic is independent of the level of noise or chosen sample size (Fig. 1*E*, right) as would be expected with normally distributed noise. That is, in data that is generated according to additivity (plus noise), the linear relationship between implicit and explicit adaptation with a slope of approximately −1 can be recovered from noisy data. This means that the 95% confidence interval of the slope can be used as a statistical test, with *α* = 0.05, of additivity of implicit and explicit adaptation.

The simulations with a generative model shown above rely on the same linear relationship between implicit and explicit adaptation as previous tests of additivity (Redding and Wallace, 1993; Sülzenbrück and Heuer, 2009; Bond and Taylor, 2015). However, previous tests only looked for significant correlations. That is; any nonflat linear relationship was taken as evidence for additivity. Here we test an additional property of data generated under additivity: a particular slope. There could be additional relevant properties (e.g., based on the intercept or residual errors) but the confidence interval for the slope of the linear relationship already provides a stricter test. We will use this approach and variations on it in several tests of the additivity assumption, both in data from our own experiment and in an aggregate data set with data from several previous papers representing various labs, setups, and paradigms.

## Varying Explicit Adaptation in Strict and Loose Additivity

If total adaptation is the sum of implicit and explicit adaptation, then, given (near) constant total adaptation, implicit and explicit adaptation should perfectly complement each other. That is, under the additivity assumption, a given change in explicit adaptation should result in a change in implicit adaptation that is of equal size, but in the opposite direction. And this should also be true across participants: a participant with higher explicit adaptation should have lower implicit adaptation and vice versa (Fig. 1). Thus, we did an experiment with three conditions that we expected to result in different levels of explicit adaptation, but highly similar total adaptation. The main condition has independent measures of implicit and explicit adaptation, and the other two set the context.

### Methods

#### Participants

For this study 72 right-handed participants (55 female; mean age, 19.8; SD, 3.7; demographics not stored for 1 participant) were recruited from an undergraduate participant pool, 24 for each of three groups (aiming, instructed, control). All participants reported having normal or corrected-to-normal vision and gave prior, written, informed consent. All procedures were approved by York University's Human Participant Review Committee.

#### Setup

**Figure 2.**
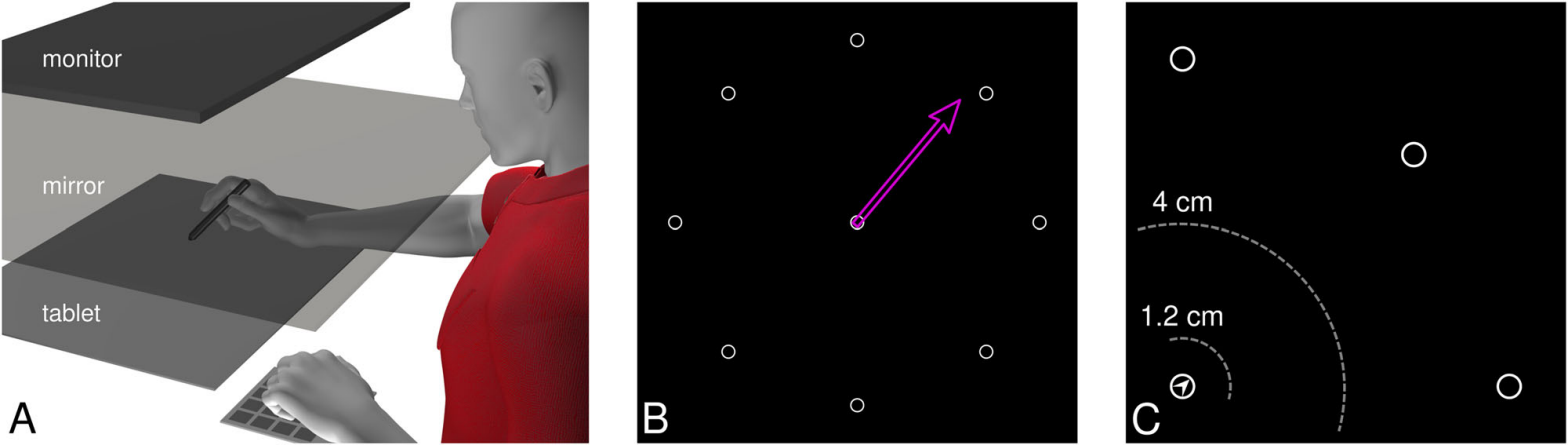
Setup, aiming and no-cursor trials. ***A***, Tablet setup with mirror. The stimuli appear in the same plane of depth as the tablet. ***B***, Aiming: the upcoming target (1 of 8) is shown with an arrow used to indicate the intended reach direction, or “aim”. ***C***, The outward reach on no-cursor trials ends when the stylus stops moving for 250 ms and is at least 4 cm away from the home position (outer gray ring). For the return home, a small arrowhead in the home position indicates at 45° intervals where the cursor is, and participants move in the opposite direction. When the stylus is then back within 1.2 cm distance of the home position, a cursor appears that needs to be moved to the home position to start the next trial.

The participants made reaching movements with a stylus on a digitizing tablet, controlling a cursor that was displayed on a downward-facing monitor (60 Hz, 20″, 1,680 × 1,050, Dell E2009Wt) seen through an upward-facing mirror in between the tablet and monitor (Fig. 2*A*). This puts the perceived stimuli in the same plane of depth as the hand, and the displacement of the cursor was scaled to match the displacement of the stylus.

##### Stimuli

The background of the screen was kept black. An open gray circle (radius, 0.25 cm) served as a home position and was located at the center of the screen (Fig. 2*B*). Eight reach targets were located at 8 cm from the start position at 0, 45, 90, 135, 180, 225, 270, and 305° and were also displayed as open gray circles (radius, 0.25 cm). A filled circle was used as a cursor (white disk; radius, 0.25 cm) on training trials. In the aiming group only (see below), an open pink arrow originating at the home position and extending 7 cm was used on aiming trials (it initially deviated ±10 or ±5° from the target, randomly chosen on each trial). On no-cursor trials a small gray arrowhead was presented within the home position, pointing to where the unseen cursor would be, in intervals of 45°, to guide participants back to the home position. More details are discussed below.

### Tasks

In each trial, participants used the stylus to move from a central home position to one of the eight targets. Before each reach, participants have to keep the cursor at the home position for 2.0 s (trials without aiming) or 0.5 s (trials with aiming). After the hold period, the target would appear and the home position would disappear, signaling to participants they should move to the target.

#### Training trials

In training trials, a cursor had to be moved from the home position to the target and back. When the cursor's center was within the radius of the target, the reach was considered ended. At that point, the target disappeared and the home position reappeared, signaling that the participant should move back to the home position. Once the cursor's center was within the radius of the home position, the trial ended. During the aligned phase, the cursor was aligned with the tip of the stylus. In the rotated phase, it was rotated 30° around the home position.

#### No-cursor trials

In no-cursor trials, the cursor disappears as soon as its center no longer overlaps with the home position. The reach is considered ended if the stylus has moved beyond 50% of the home–target distance (4 cm; Fig. 2*C*) and there is no movement for 250 ms (or the total movement during the last 250 ms is <1% of the home–target distance). At this point, the target disappears, the home position reappears, and an arrow at the home position indicates where the (rotated) cursor would be relative to the home position (in increments of 45°). Participants use this to move back toward the home position, and when the cursor position is within 15% of the home–target distance (1.2 cm; Fig. 2*C*), the cursor is shown again, to get back on to the home position.

In the rotated phase, these were used in a PDP (Jacoby, 1991; Werner et al., 2015) in all three groups. In this PDP, people reach for a target without cursor, while either including or excluding their strategy (see below). The “exclude” strategy blocks are a measure of implicit adaptation, while the include strategy blocks should measure both implicit and explicit adaptation (Werner et al., 2015; Modchalingam et al., 2019; Gastrock et al., 2020; Maresch et al., 2021b). If implicit and explicit adaptation are additive, then subtracting reach deviations measured in include strategy blocks from reach deviations measured in exclude strategy blocks should be a measure of explicit adaptation. Whether or not this is true, if the responses are different in the two blocks, then participants can dissociate their strategic responses from unconscious adaptation, which shows that some degree of explicit adaptation is occurring. Note that if there is no difference in the responses, this is highly suggestive that no explicit adaptation is occurring, but not definitive proof.

#### Aiming

Before training trials, participants in the aiming group would see the upcoming target, and they could orient an arrow using the arrow keys on an extra key pad, to indicate the direction in which they would move their hand in order to get the cursor to the target. In the aiming group, the training blocks are called “Aim and reach” blocks, and the instructions they were given include the following: “Point the arrow as accurately as you can to indicate the movement you want your hand to make, so that you can get the cursor on the target.”

### Instructions

#### Strategy

Participants in the instructed group received detailed instructions in between the aligned and rotated phase, while participants in the other groups had a break of about the same duration. The instruction included an animation of the perturbation and explanation of a strategy to counter the perturbation (move in direction rotated 1 h on a clock face). They were tested on understanding the strategy by drawing reaches on a clock face toward targets in all quadrants.

All groups were informed that the way the cursor moves changes in the next part of the experiment, and they were told that they have to figure out how to deal with this and that they should remember what their strategy is, since they will be asked to reach while using this strategy and while not using this strategy, with this instruction:

“This is part 2 of the experiment. For these coming tasks, the filled cursor will move a bit differently, and you will need to compensate for this. However you compensate, keep that strategy in mind since you will be asked to use this strategy several times, when reaching without a cursor. Sometimes you will be asked to NOT use this strategy when reaching without a cursor.”

These instructions were read to participants before the rotated part of the task.

#### Process Dissociation Procedure

At the end of the rotated session, participants do no-cursor reaches in two kinds of blocks: either while including or excluding their strategy, with these instructions:

Include: “For THESE trials, make full use of any strategies you learned just now.”

Exclude: “For THESE trials, do not make use of any strategies you learned earlier and treat this as you did the original ‘*reach for target*’ task in part 1.”

Importantly, the instructions about what to do in the PDP blocks were identical for all groups, such that they can not explain any differences in performance.

### Procedure

**Figure 3.**
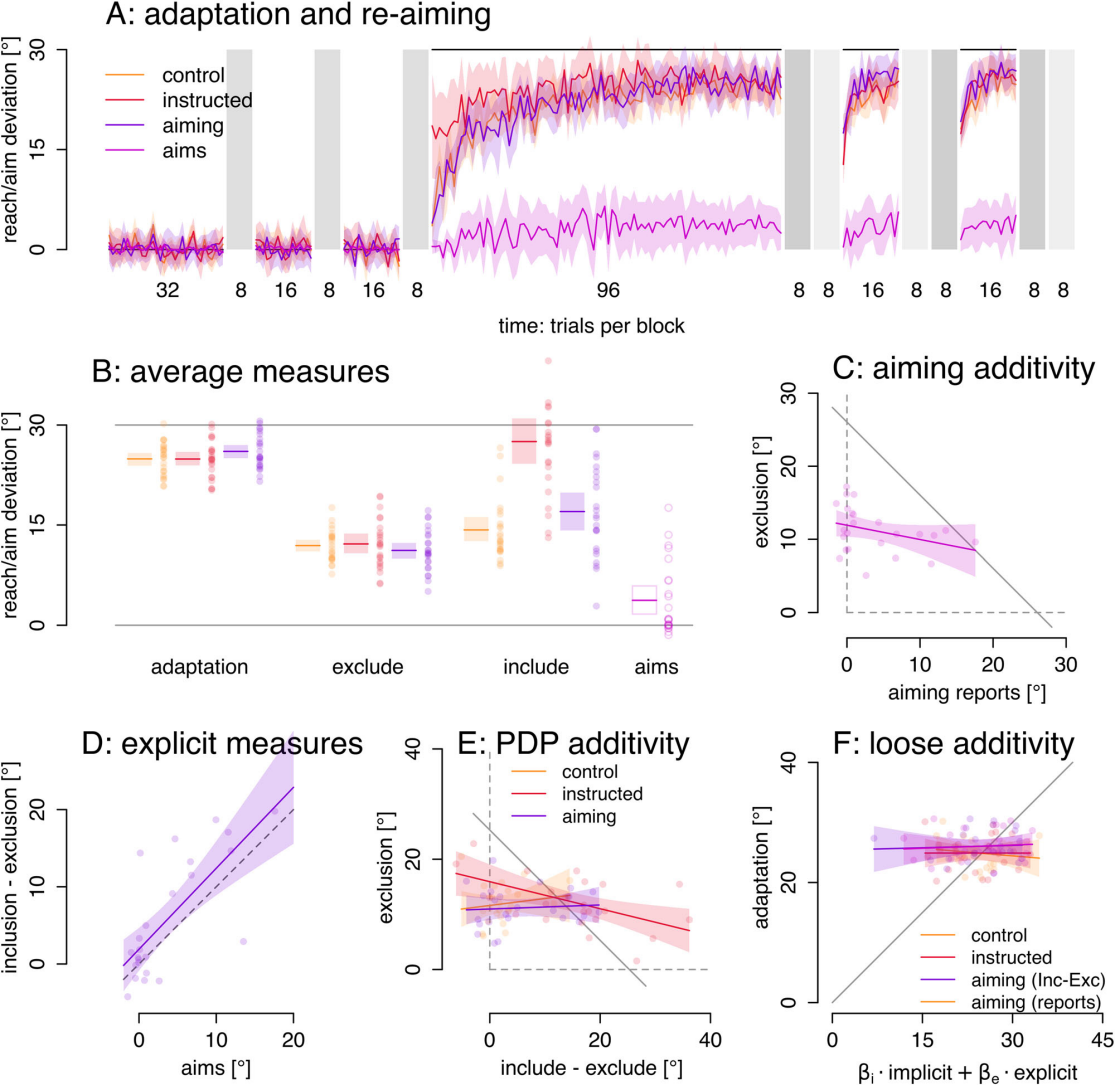
Adaptation measures and additivity. Areas in color denote 95% confidence intervals of the mean. Dots are individual participants. ***A***, Adaptation and aiming responses throughout the perturbation schedule. No-cursor blocks in gray with inclusion and exclusion trials in the rotated phase. ***B***, Averaged estimates of adaptation, exclusion, and inclusion and aiming reports. ***C***, Strict additivity would predict that exclusion scores can be predicted from aiming scores and total adaptation and would lie close to the gray diagonal (slope −1), but there is close to no relationship between directions of aiming responses and exclude strategy reaches. ***D***, Linear regression shows that aiming reports and include–exclude difference scores agree fairly well, such that we may be able to use include–exclude difference scores as a stand-in for aiming responses. ***E***, Using indirect measures of explicit adaptation, we also can not confirm strict additivity, which would predict data lie close to the gray diagonal (with slope −1). ***F***, Loose additivity would allow a weighted sum to predict total adaptation, with data over predictions on the gray unity line, but in this data set there is no relationship.

All participants performed 264 reaching trials of various kinds (Figs. 2, 3), organized into blocks of 8 trials. In each block, all eight targets were used in a randomized order. The task started with an aligned phase with first 32 training trials and three blocks of no-cursor trials (Fig. 3, gray rectangles), with two blocks of training trials before the second and third no-cursor block. In the rotated phase, a rotation of 30° is applied to the cursors (Fig. 3, black lines indicate perfect reaches). These included 96 training trials, followed by three sets of two no-cursor trial blocks. Adaptation is “topped up” before the second and third of these sets of no-cursor trials with 16 rotated training trials. The no-cursor trials in the rotated part are divided into two blocks: one where participants are instructed to include their strategy and one where they do not include their strategy. In one group, the “aiming” group, every training trial is preceded by aiming: participants are shown the upcoming target and are asked to indicate where they will move their hand in order to get the cursor to the target. In the other groups, participants wait 1.5 s instead of aiming, to keep the total time roughly equal.

Participants were intermittently monitored during the experiment and encouraged to make straight, smooth reaches, to faithfully do no-cursor reaches according to instructions and to really consider where the aiming arrow should be pointed, if experimenters suspected the participant not to perform optimally.

### Analyses

For all reaches (both training and no-cursor trials), we calculated the angular reach deviation at one-third of the home–target distance (∼2.67 cm) and similarly used the aiming arrows angular deviation for comparison. We remove training reaches and reaiming responses deviating >60° from the target (controls, 0; instructed, 1; aiming, 3; reaiming responses, 0) and subsequently all angular deviations outside the average ±3 standard deviation for each trial across participants within the group (controls, 15; instructed, 10; aiming, 12; reaiming responses, 30). The maximum number of data points removed for any participant was 6 (control, 3; instructed, 4; aiming, 6; reaiming responses, 6), and many participants had no trials removed (control, 16; instructed, 19; aiming, 16; reaiming responses, 12). No participants were removed, and no trials were removed from no-cursor data.

We use the average reach deviation in the last eight trials before each of the no-cursor blocks and subtracted the average in the aligned phase from the average in the rotated phase to assess final adaptation. In the aiming group, we used the same blocks to assess final reaiming. We use all the include/exclude strategy no-cursor trials to assess implicit and explicit adaptation using the PDP. We subtract reach deviations from the aligned part of the task from the same measures obtained in the rotated part of the task to correct for baseline biases. This way we obtain adaptation in the training trials, with and without strategy no-cursor reach deviations and reaiming in the aiming trials. All of these are based on an equal number of trials and taken from parts of the experiment that are as similar as possible.

We compare the exclusion reach deviations as a measure of implicit adaptation and the final, total adaptation between the three groups. To assess explicit adaptation, we then compare the difference between inclusion and exclusion reach deviations. Within the aiming group we also compare the difference between inclusion and exclusion reach deviations, with the final aiming responses, as measures of explicit adaptation. All these allow testing of additivity on estimates of common measures of implicit and explicit adaptation processes.

#### Strict linear additivity

We test additivity in two approaches. First, we investigate the notion total adaptation is simply the sum of explicit and implicit adaptation (Redding and Wallace, 1993; compare Fig. 1):

A=I+E.

In particular, the way it is sometimes used to determine implicit adaptation is as follows:

I=A−E,

which is equal to a linear model, expressing the relationship between implicit and explicit adaptation across participants (p):

$$I_{p}=\beta_{0}A_{p}+\beta_{1}E_{p},$$

where $\beta_{0}A$ is the intercept and $\beta_{1}$ is the slope $\left(\beta_{0}=1,\;\beta_{1}=-1\right)$. It simply means that, with no explicit adaptation, implicit adaptation is equal to total adaptation (the intercept), and with every increase in explicit adaptation, there is an equally sized decrease in implicit adaptation (slope of −1; Fig. 1*D*,*E*). This linear model directly follows from the additivity assumption and would apply both within and across participants. A simulation to recover the slope of −1 from data generated according to this model plus noise shows that the 95% confidence interval of the fitted slope includes −1 in ∼95% of simulations (Fig. 1*E*). That is, if we decide that actual data is in accordance with additivity when the 95% confidence interval of the slope of a linear model predicting implicit from explicit adaptation includes −1, this is equivalent to a NHST statistic with *α* = 0.05. Hence, we will use this statistical approach.

#### Loose linear additivity

To account for some systematic misestimates in either implicit or explicit adaptation, we use a second, less strict, model of additivity. Here, we predict total adaptation by summing weighted versions of implicit and explicit adaptation across all participants in each group:

$$A_{p}=\beta_{0}I_{p}+\beta_{1}E_{p}.$$

If this model of loose additivity works, then there should be a linear relation between predicted and actual adaptation with an intercept of 0 (no intercept) and a slope of 1. And here we test this by fitting a linear model on real adaptation over predicted adaptation, and we consider the model confirmed if the 95% confidence interval for the slope includes 1.

### Results

In our experiment, three groups of participants adapted to a 30° rotation: the main “aiming” group (*N* = 24) that reported their aiming before every training trial by orienting an arrow in 1° steps, as well as an “instructed” group (*N* = 24) that was made aware of the rotation and given a counterstrategy, and a “control” group (*N* = 24). All groups repeated counterbalanced blocks of include and exclude strategy no-cursor reaches (Werner et al., 2015) to assess implicit and explicit adaptation. Aiming reports are used as an independent measure of explicit adaptation. See Methods for more details.

First, we find no group difference in adaptation (Fig. 3*A*,*B*; *F*_(2,69)_ = 1.30; *p* = 0.28; *η*^2^ = 0.036; BF_10 _= 0.316) or in exclusion as measure of implicit adaptation (Fig. 3*B*; *F*_(2,69)_ = 0.59; *p* = 0.555; *η*^2^ = 0.017; BF_10 _= 0.185). Additivity applied to the PDP measures would then predict equal inclusion deviations in the three groups (inclusion = implicit + explicit), but we find that the instructed group has higher inclusion deviations (*F*_(2,69)_ = 24.72; *p* < 0.001; *η*^2^ = 0.417; BF_10 _= 1,369,327), with no difference between the other two groups. That is, the group averages of measures with built-in additivity do not seem to show additivity.

However, additivity should be tested by comparing the relationship between independent measures of explicit and implicit adaptation across various measures, which we do in the “aiming” group. We can see that the data does not appear to lie along the gray diagonal (Fig. 3*C*) that depicts additivity based on the average total adaptation (compare Fig. 1*D*). Average adaptation in the aiming group is 26.0° (range, 21.5–30.6°; SD: 2.6°). Aiming response range from −1.4 to 17.5° with an average of 3.7°, and exclude strategy reach deviations range from 5.1 to 17.2° with an average of 11.2°. A linear regression of exclusion scores over aiming responses (Fig. 3*C*; *F*_(22,1)_ = 2.8; *p* = 0.11; *R*^2^_adj _= 0.073) has a slope of −0.196, and the 95% confidence interval of that slope (−0.437, 0.046) does not include −1, though it does include 0. That is, using independent measures of implicit and explicit adaptation, we can not find evidence for the additivity assumption.

Perhaps the difference between include and exclude strategy reach deviations are an acceptable stand-in for a measure of explicit aiming, such that we can test additivity in the other two groups. We test this in the aiming group, by predicting the inclusion–exclusion difference scores from the reported aims, with a linear regression. The two measures largely agree (Fig. 3*D*; *F*_(1,22)_ = 25.84; *p* < 0.001; *R*^2 ^= 0.5192; intercept = 1.9; slope = 1.05; slope 95% CI: 0.62–1.47)—in this data set, but not everywhere (Heirani Moghaddam et al., 2021; Maresch et al., 2021b). Given this, we also test strict additivity in the three groups using the inclusion–exclusion differences as a measure of explicit adaptation (Fig. 3*E*) but find that in none of the three groups, the 95% confidence interval of the slope includes −1 (aiming, −0.17–0.24; control, −0.26–0.51; instructed, −0.41 to −0.08). This result does rely on the additivity assumption, and yet it shows no relationship between implicit and explicit adaptation. However, the finding based on aiming reports uses independent measures and should be considered more reliable.

## Do the Fast and Slow Processes Map onto Measures of Explicit and Implicit Adaptation?

Next, we test a particular application of the additivity assumption, in a common state-space model with two adaptive processes. In this model, the so-called “slow” and “fast” processes are strictly additive (Smith et al., 2006) and are sometimes assumed to map onto implicit and explicit adaptation, respectively (McDougle et al., 2015). In previous work (McDougle et al., 2015), the similarity between averaged aiming reports and the fast process fitted to group data is striking. However, given that the model also assumes additivity and that the measure of explicit adaptation matches the model's fast process fairly well, it has to be the case that the subtractive estimate of implicit adaptation matches the slow process equally well. That is, while the literature supports a link between the model's fast process and an independent measure of explicit adaptation, the link between the slow process and implicit adaptation is fully dependent on the largely untested assumption of strict linear additivity and should not be relied upon, indiscriminately. We test this here in the data from the aiming group in the experiment described above.

### Methods

#### Two-rate model

Using data from the experiment described above, we test a state-space model with a strong additivity assumption (Smith et al., 2006) and two learning processes that are sometimes assumed to map onto implicit and explicit adaptation (McDougle et al., 2015). The model posits that motor output on trial *t* (*X_t_*) is the straightforward, unweighted sum of the states of a slow and fast learning process:

$$X_{t}=F_{t}+S_{t},$$

where the states of both the fast and slow process are each determined by a learning rate (*L*) applied to the error on the previous trial and a retention rate (*R*) applied to the state of each process on the previous trial:

$$F_{t}=L^{f}\cdot E_{t-1}+R^{f}\cdot F_{t-1},$$

and

$$S_{t}=L^{s}\cdot E_{t-1}+R^{s}\cdot S_{t-1}.$$

This is further constrained by $L^{s}<L^{f}$, and $R^{f}<R^{s}$.

##### Parameter recovery simulation

The model typically is fit on data with a different perturbation schedule, but the two blocks of no-cursor reaches causing some decay in learning, followed by relearning, may suffice for a reasonable group-level fit. To test this, we ran a parameter recovery simulation for the model using the training reach deviations in the control group (fit model 1,000 times to data simulated as original model + random noise drawn from a normal distribution with mean = 0 and a standard deviation equal to the square root of the MSE of the original model fit). This shows that the parameters can be recovered quite well (not shown, bootstrapped data on OSF: https://osf.io/3yhw5). For each parameter, the originally fitted value falls in the 95% interval of the recovered values (*L^s^*: 0.028 < 0.035 < 0.043; *L^f^*: 0.087 < 0.105 < 0.129; *R^s^*: 0.991 < 0.994 < 0.996; *R^f^*: 0.832 < 0.885 < 0.912). This means that fitting the model to reach deviations in this perturbation schedule is robust, at the group level (Albert and Shadmehr, 2018), and we can use the model to test if its processes map onto the measures of explicit and implicit adaptation throughout learning.

##### Aware and unaware learners

**Figure 4.**
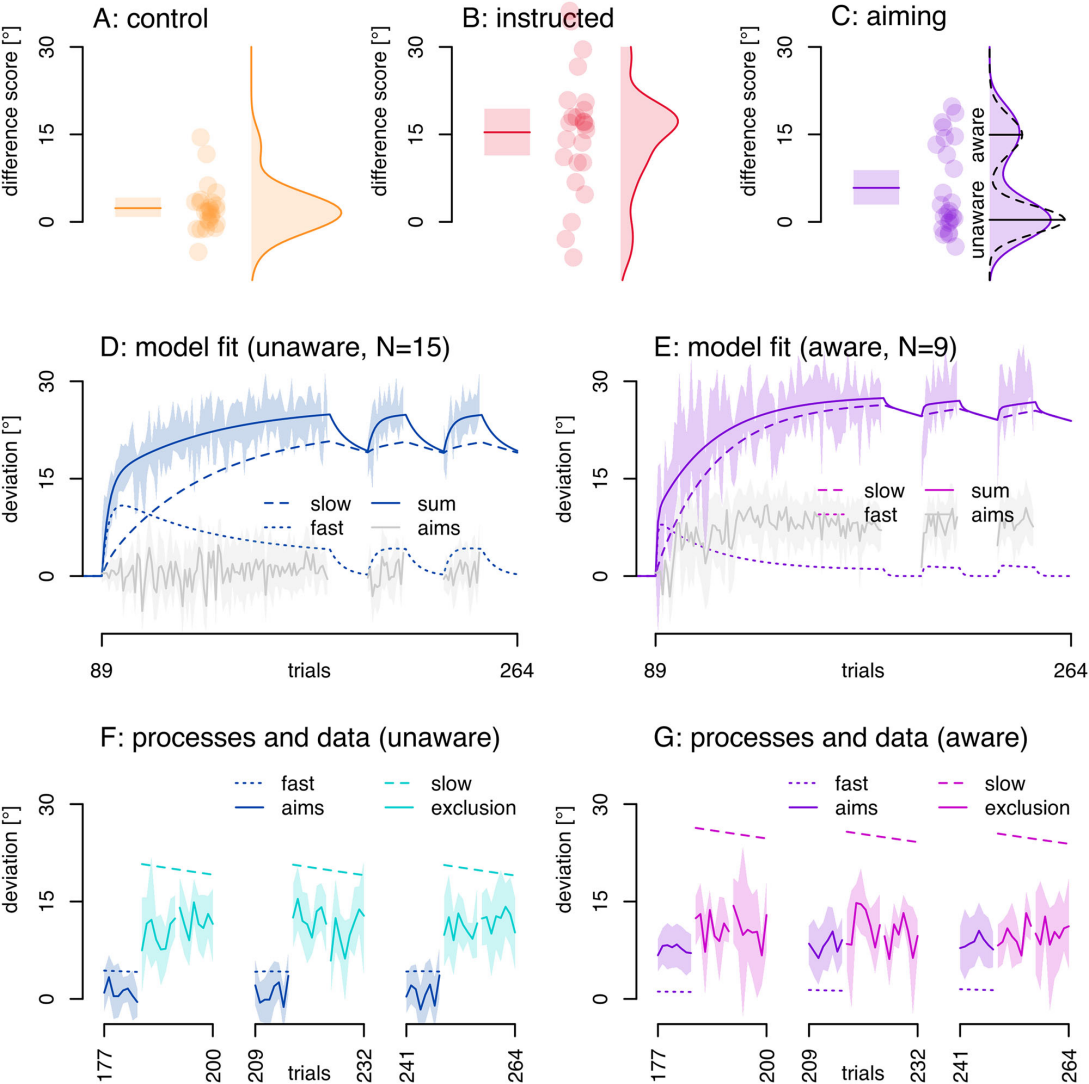
Additive state-space model. ***A–C***, Distributions of differences between include and exclude strategy reach deviations, depicted as density plots. Dots represent individual participants; solid lines and shaded regions in bar plots represent the mean and 95% confidence interval. ***C***, In the aiming condition, the distribution is bimodal (*p* < 0.001) suggesting aware and unaware subgroups. The underlying processes in the two-rate model fits should differ between these two subgroups if the fast process maps onto explicit adaptation. Thus, we fit the model separately for each subgroup (***D***, ***E***). ***D***, ***E***, Shaded area represents the 95% confidence interval of reach deviations and the solid line the two-rate model fitted to these reach deviations, for the unaware aimers (***D***) and the aware aimers (***E***). ***F***, ***G***, Two-rate model processes compared with data. In the unaware subgroup (***F***), the confidence interval of the aiming responses (jagged continuous dark blue line with 95% confidence interval) is near zero and excludes the fast process (smooth, dotted, dark blue line) on most trials. In the aware subgroup (***G***), the fast process (smooth dotted purple line) for the most part is outside the 95% confidence interval of the aiming responses (jagged continuous purple line with 95% confidence interval). Similarly, the slow process of each subgroup's model fit (the dashed lines) are above the 95% confidence interval for the exclude strategy reach deviations (jagged continuous lines in the no-cursor blocks: light blue for the unaware aiming group, light pink for the aware aiming group). Thus, in both subgroups, the fast process does not predict aiming responses, and the slow process does not predict strategy exclusion reach deviations.

On average, we find only a small reaiming strategy in the aiming group (Fig. 3*B*: 3.74°; *t*_(23)_ = 3.38; *p* = 0.001; *η*^2 ^= 0.497). However, we observed that the distribution of the differences between inclusion and exclusion reach deviations for participants in the aiming group is bimodal (relative log-likelihood based on AICs of unimodal distribution compared with bimodal: *p* > 0.001). The two peaks are close to the average inclusion–exclusion differences in the control and in the instruction group (Fig. 4*A–C*). This likely indicates that for 9/24 participants, the addition of aiming had the same effect as instructions, but no effect for the remaining 15 participants.

We split the aiming group into aware aimers and unaware aimers, as these subgroups should have different levels of explicit adaptation. We fit a separate two-rate model to the averaged training reach deviations in the aware aimers as well as those in the unaware aimers. We then test whether or not the fast process falls in the 95% confidence interval of each subgroups’ aiming responses and whether or not the slow process falls in the 95% confidence interval of exclusion reach deviations in each subgroup. The comparison between the slow process and exclude strategy reach deviations (implicit) can only be done in the no-cursor blocks, when the processes have presumably saturated. The comparison between aiming responses and the fast process is then also done when the processes have saturated: during the three blocks of 8 trials just before the no-cursor blocks. If the model processes fall within the 95% confidence interval of the data, this would support additivity as implemented in the two-rate model.

### Results

We test if the state-space model's slow process maps onto exclusion trials (implicit) and if the fast process maps onto aiming responses (explicit). We separately fit the state-space model to the averaged reach deviations in aware and unaware aimers (Fig. 4*C*). In the aware aimers (Fig. 4*D*, purple), the fast process (purple smooth continuous line) is lower than aiming reports (purple jagged line with 95% confidence interval) and vice versa in the unaware aimers (dark blue). In both subgroups the 95% confidence interval of the aiming data excludes the fitted fast process (except for a few trials at the start). In both subgroups the slow process (dashed lines) is higher than exclusion trials during all no-cursor blocks (95% CIs of data exclude model processes; pink, aware aimers; light blue, unaware aimers), confirming that the slow process does not capture implicit adaptation (Ruttle et al., 2021; Bansal et al., 2023). That is, the strictly additive processes of the state-space model do not align with direct measures of implicit and explicit adaptation in this data set.

## Testing Additivity across the Field

While it stands to reason that adaptation is larger when both implicit and explicit adaptation processes contribute (Mazzoni and Krakauer, 2006; Benson et al., 2011; Neville and Cressman, 2018), few studies indicate some form of additivity (Sülzenbrück and Heuer, 2009; Bond and Taylor, 2015). Other studies do not show additivity (Werner et al., 2015; Schween et al., 2018; Modchalingam et al., 2019; Gastrock et al., 2020) or they show partial additivity or additivity only in some participants (Neville and Cressman, 2018; Bromberg et al., 2019) or it is unclear to us whether or not the data support additivity (Heirani Moghaddam et al., 2021; Maresch et al., 2021b). So far, we rejected additivity based on one data set only. It would be easy to dismiss these findings as a one-time occurrence. However, if additivity does not hold, this has implications for the field. Hence, we test strict and loose linear additivity in a number of other data sets, with independent if not simultaneous measures of explicit, implicit, and total adaptation. We collected data sets from previous work, both from other labs and our own, to test strict and loose additivity beyond the single experiment described above with data representing various approaches in the field.

### Methods

We either asked the original authors (Taylor et al., 2014; Bond and Taylor, 2015; Brudner et al., 2016; Neville and Cressman, 2018) or downloaded data from public repositories (Modchalingam et al., 2019, 2022; Maresch et al., 2021b; Decarie and Cressman, 2022), and used one unpublished data set sent to us by Dr. Jordan Taylor as well as one incomplete data set from study conducted in our lab (D’Amario et al., 2024). This is not an exhaustive literature search but should represent several types of equipment, approaches, and research groups, and it should increase power. See Table 1 for more information.

**Table 1.** Sources of external data

Study	Data source	Adaptation	Implicit	Explicit
Sources with independent aiming and exclusion				
Taylor et al. (2014); Bond and Taylor (2015); Brudner et al. (2016); unpublished data set	Private exchange	Last 16 training trials	Starting value of an exponential function fitted to the first 16 washout trials	Last 16 aiming reports of training
Schween et al. (2018 exp. 1 & 2; Schween et al., 2019 exp. 1, 2 & 3)	Private exchange	Average across 9 targets	Average across targets straddling the averaged generalization curve's peak	Average across 9 targets
D'Amario et al. (2024)	In-lab exchange	16 training trials	16 exclusion trials	8 aiming trials
Sources with PDP difference scores				
Neville and Cressman (2018)	Private exchange	Last 9 training trials	9 exclusion trials in in last prepause PDP block	9 inclusion–9 exclusion trials in last prepause PDP block
Modchalingam et al. (2019)	OSF repository linked in paper: https://osf.io/mx5u2/	Last 18 trials of the first rotated training	4 × 9 exclusion trials	4 × 9 inclusion–4 × 9 exclusion trials
Maresch et al. (2021b)	OSF repository linked in paper: https://osf.io/6yj3u/	Last 8 trials in “top-up” block	Last 16 exclusion trials	Last 16 inclusion–last 16 exclusion trials
Decarie and Cressman (2022)	Supplementary file with paper: https://link.springer.com/article/10.1007/s00221-022-06352-4	Last 15 trials of rotated reach training	Last 6 exclusion trials	Last 6 inclusion–last 6 exclusion trials
Modchalingam et al. (2023)	https://osf.io/a7k6s/	2 × 9 training trials before PDP blocks	2 × 9 exclusion trials	2 × 9 inclusion–2 × 9 exclusion trials

The number of trials are indicated, measures are from trials when adaptation has saturated, and measures should be stable, so they can be averaged for more precision. The exceptions are the data sets from the papers in the first row, where measures of implicit adaptation are taken during its washout. Here we fit an exponential function to the first 16 trials, and use the starting value as an estimate of implicit adaptation.

#### Normalization

**Figure 5.**
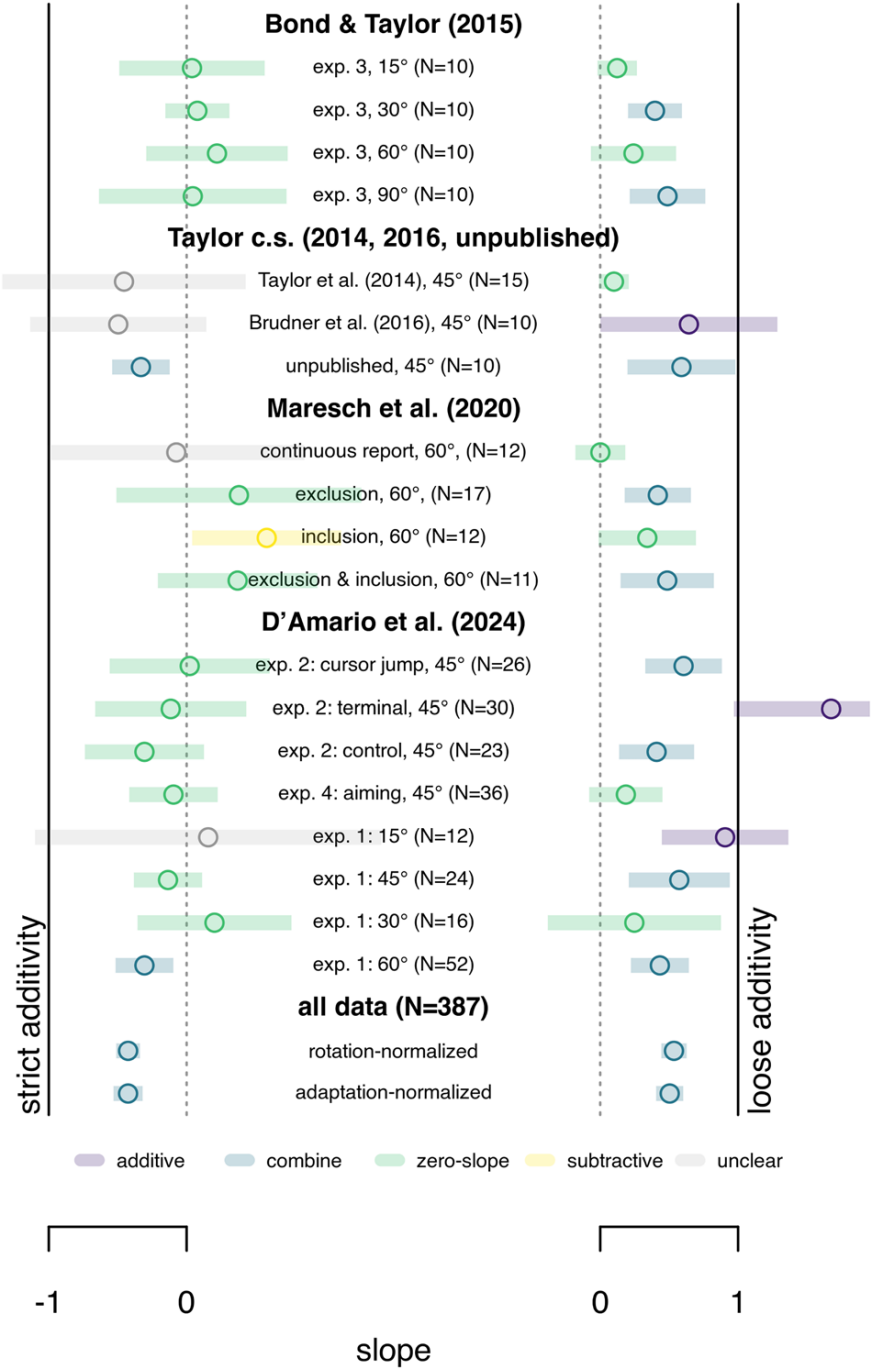
Additivity in a cross section of studies with independent measures of implicit and explicit adaptation. In each group (rows) we test strict additivity (left) by testing if −1 (continuous black line) is included in the 95% confidence interval (colored bars) of the slope of a linear model fitted to independent measures of both implicit and explicit adaptation. No groups demonstrate strict additivity. We also test loose additivity (right), where in each data set a free weight is set to both implicit and explicit contributions before summing them to predict total adaptation. Here the 95% confidence interval (colored bars) of the slope of predicted over actual adaptation should include 1 (continuous black line). Only 5 of 24 subsets show loose additivity. In the combined data (bottom two rows), there does seem to be support for some form of combining explicit and implicit adaptation.

As before, we test if the confidence intervals for the slopes of the strict and loose models of linear additivity include −1 and 1, respectively, both on all individual data sets, and all the data combined. The rotations varied between 15 and 90°, so we need to normalize the measures to be able to compare data across groups. Since the additivity assumption predicts adaptation (not rotation), we normalize relative to each participants’ estimate of total adaptation. If additivity holds, the normalized measures of implicit and explicit adaptation should then sum to 1. We do test normalization by rotation on all data combined as well, and this yields highly similar results (Fig. 5, last two rows). However, to stay more in line with the tested additivity assumption, we use only the adaptation-normalized scores for the further exploration of aggregate data.

### Results

The literature is divided into the additivity assumption of implicit and explicit adaptation (Maresch et al., 2021a). That's why we reanalyzed data from 11 studies (Taylor et al., 2014; Bond and Taylor, 2015; Brudner et al., 2016; Neville and Cressman, 2018; Schween et al., 2018, 2019; Modchalingam et al., 2019, 2022; Decarie and Cressman, 2022; D’Amario et al., 2024). There are 24 groups (*N* = 488) with independent measures of implicit and explicit adaptation, and 16 groups (*N* = 325) using the PDP, that has an independent measure of implicit adaptation, but not of explicit adaptation. For every participant we took an average across multiple trials at the end of training to estimate explicit, implicit, and total adaptation when each of these measures should have saturated [the “stepwise” participants (Modchalingam et al., 2022) are assessed four times, at four different rotation sizes, for 43 “groups” total].

First, we look at data with truly independent measures of explicit and implicit adaptation (Table 1, top rows; Fig. 5). We divide each participants’ estimates by their total adaptation and for each subgroup accept strict additivity (Fig. 5, left) or loose additivity (Fig. 5, right) if the 95% CI for the slope of the model includes −1 or 1, respectively (as above; Figs. 1*D*,*E*, 3*D*,*E*). None of the groups support strict additivity (Fig. 5), and only 5 of 24 subgroups support loose additivity. Consequently, in all participants combined (Fig. 5, bottom two rows), there is no support for additivity either.

**Figure 6.**
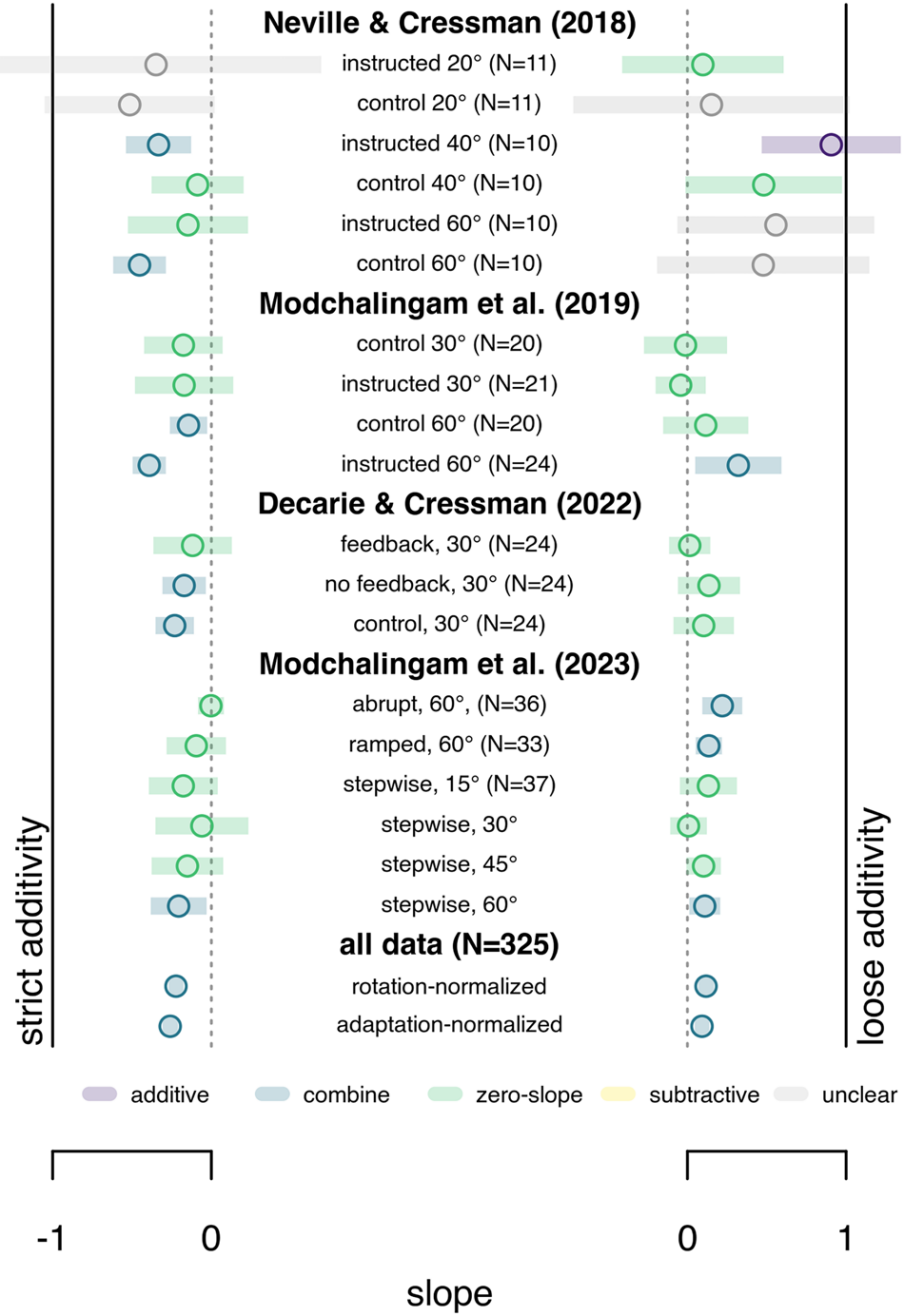
Additivity in a cross section of studies where implicit and explicit adaptation are not independent. All data comes from studies using PDP. Here total adaptation is not relied on, as in most studies using measures of aiming, but the measures of explicit and implicit adaptation are related (see Methods). Strict additivity (left) is supported if the 95% confidence interval (colored bars) of the slopes fitted to implicit over explicit adaptation include −1 (continuous black line). Loose additivity (right) is supported if the 95% confidence interval (colored bars) of a line fitted to the predicted over the measured adaptation includes 1 (continuous black line). No groups support strict additivity, and only 1 of 19 supports loose additivity. The overall relationship between implicit and explicit adaptation (bottom rows) is weaker than in studies with independent measures (Fig. 5), despite the measures used here relying on a form of additivity.

We also test the relationship between estimates of implicit and explicit adaptation, when these are not strictly independent (Table 1, bottom rows; Fig. 6). Here, this coincides with studies using the PDP only (or some variant), where explicit adaptation is taken to be the difference between inclusion and exclusion trials, while implicit adaptation is independently estimated by exclusion trials. That is, the additivity assumption is built into the estimate of explicit adaptation. Despite this, however, none of the 19 groups support strict additivity, and only 1 supports loose additivity.

## Maximum Likelihood Estimation of Adaptation

**Figure 7.**
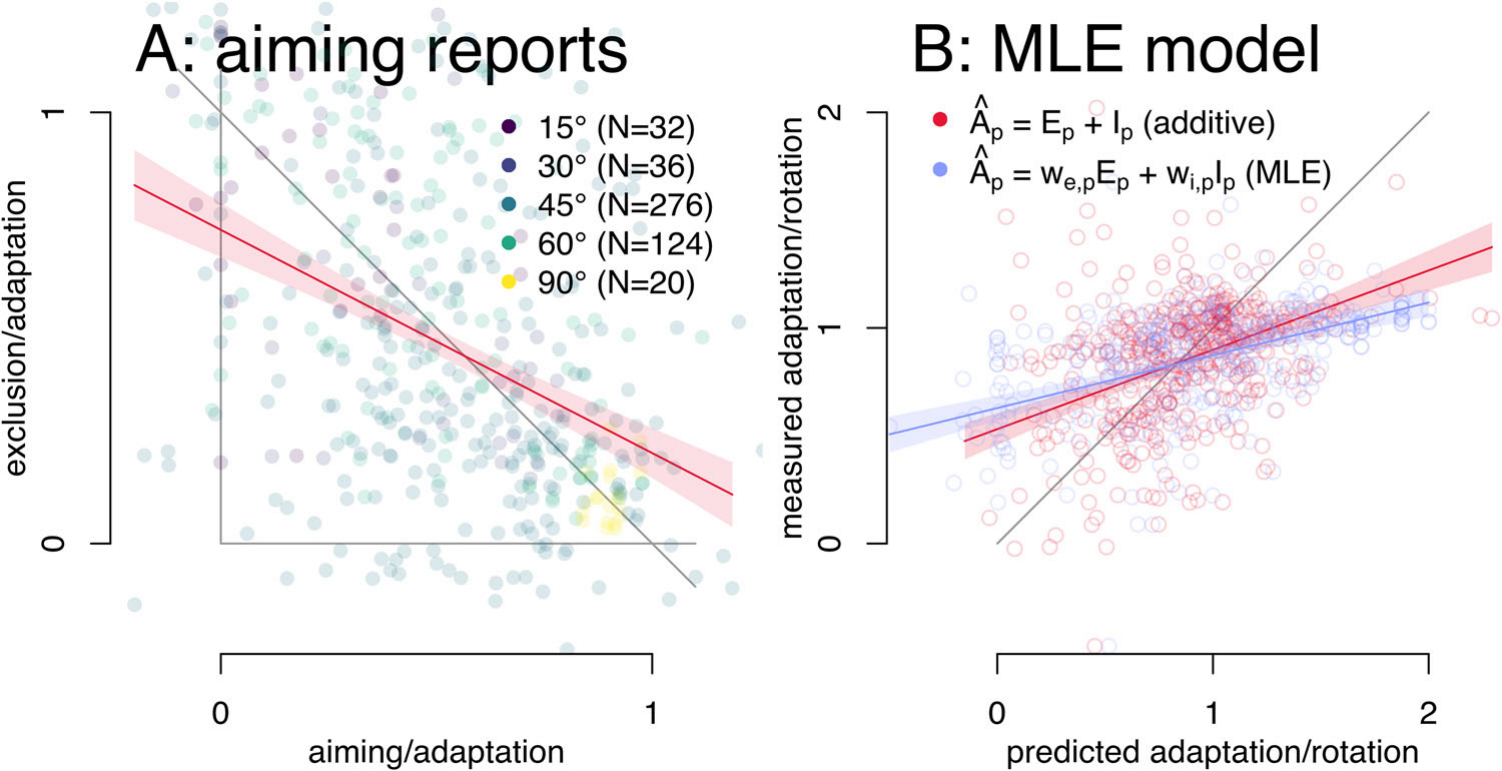
MLE combining implicit and explicit adaptation. ***A***, Adaptation-normalized data and a linear regression of implicit over explicit adaptation (*N* = 488). The gray diagonal indicates perfect additivity. ***B***, Predicted adaptation based on strict additivity and on MLEs for 387 participants. The gray diagonal indicates perfect prediction.

The aggregate data does show a combined effect: slopes are different from 0 (Figs. 5–7*A*). If contributions from implicit and explicit adaptation are not added, the question is: how are they combined? We test one possible answer, inspired by maximum likelihood estimation, using the collection of data sets from above. In this approach adaptation is estimated by weighting explicit and implicit adaptation by the inverse of their variance. That is, the more reliable adaptation process contributes most to total adaptation. Similar approaches provide powerful explanations for combined sensory estimates, so this may be a more biologically plausible mechanism.

### Methods

#### Maximum likelihood estimates

To test if implicit and explicit adaptation are combined based on their relative reliability, with a greater contribution for the more reliable process, we applied a maximum likelihood estimate (MLE). Here each of the processes is weighted by the relative inverse of their variance within each participant:

$$w_{i,p}=\frac{1/\sigma_{i,p}}{\left(1/\sigma_{i,p})+(1/\sigma_{e,p}\right)},$$

and

$$w_{e,p}=\frac{1/\sigma_{e,p}}{\left(1/\sigma_{i,p})+(1/\sigma_{e,p}\right)},$$

where $w_{i,p}$ and $w_{e,p}$ are the weights for implicit and explicit adaptation, respectively, for participant p and $\sigma_{i,p}$ and $\sigma_{e,p}$ are the variance of implicit and explicit measures for participant p. These are based on the data but require a direct measure of each. This is only possible for independent measures of implicit adaptation (such as exclusion trials) as well as for explicit adaptation (such as reaiming) with a fair number of individual trials available. This restricts us to data from four papers (Taylor et al., 2014; Bond and Taylor, 2015; Brudner et al., 2016; Maresch et al., 2021a) plus one unpublished group, and the incomplete data set from our lab, for 488 participants adapting to rotations of 15, 30, 45, 60, and 90° (Fig. 7*A*). To account for the different rotation sizes, we use rotation-normalized estimates of implicit, explicit, and total adaptation. The estimates of total adaptation for each participant p are then the following:

$$A_{p}=w_{i,p}E_{p}+w_{e,p}I_{p},$$

in the MLE and simply

$$A_{p}=E_{p}+I_{p},$$

for the comparable additive model.

To be clear, in each case $A_{p}$ is the predicted adaptation for participant p, based on $E_{p}$ and $I_{p}$, the average measure of explicit and implicit adaptation for the same participant.

The weights in MLEs sum to 1, but in the strictly additive approach, they would sum to 2. We also tested a sum of weights of 2, but this results in a slightly worse fit. We tested some other variants as well: a freely fitted sum of weights, adding a constant, or scaling the contributions from implicit and explicit adaptation independently. These all produce comparable results, so we opted for presenting the simplest model.

### Results

Some models of adaptation use Bayesian mechanisms to weigh different contributions (Berniker and Kording, 2011; Tsay et al., 2022), and these mechanisms are not linearly additive, so here we test one as an alternative way explicit and implicit adaptation are combined. In particular we test a MLE (Ernst and Banks, 2002). The weight applied to the average of each contribution is the relative inverse of its respective variance for every participant which means that a separate estimate of variance of both implicit and explicit adaptation is required (details in Methods). This is only possible in 488 participants, and we test how well a strictly additive and an MLE mechanism predict the measured adaptation. As can be seen (Fig. 7*B*), neither predicts adaptation very well, and the maximum likelihood approach does slightly worse than the additive approach. That is, the maximum likelihood estimation is an unlikely alternative to strict additivity. The way implicit and explicit contributions are combined in motor adaptation remains unclear.

## Discussion

In this study we tested whether measures of implicit and explicit adaptation linearly add in total adaptation. In particular, we tested the validity of deriving an indirect, “quasi” measurement of implicit adaptation by subtracting a measure of explicit adaptation from a measure of total adaptation. It seems clear that explicit and implicit processes interact to determine the amount of total adaptation, but we find no evidence supporting linear addition. Below, we discuss what this means for our understanding of explicit and implicit adaptation, implications for future practice, as well as the interpretation of some previous findings.

While both implicit and explicit processes contribute to adaptation, and a plethora of work has been done testing what either process is sensitive to, there are two main sources of problems with how we understand the contribution of each. First, the methods we use to measure both implicit and explicit adaptation all have their issues (Maresch et al., 2021b). The second source is the focus here; we do not know the neural mechanism by which contributions from different learning processes are combined in behavior.

In our experiment, as well as in a cross section of other data, there was unexpectedly little evidence of an additive relationship between independent measures of explicit and implicit adaptation. This means that implicit adaptation can not be estimated by subtracting explicit adaptation from total adaptation or vice versa. Previous work on visuomotor adaptation might need to be re-evaluated if their findings on implicit adaptation rely on a strict additivity assumption, e.g., when subtracting a measure of explicit adaptation from total adaptation to get a measure of implicit adaptation (usually in studies relying on measures of reaiming). Conversely, explicit adaptation is sometimes gauged by subtracting reach deviations in exclusion trials from those in inclusion trials (as we did here). This is done in studies relying on the PDP, although this appears less common. Nevertheless, it should be clear that this approach suffers from the same lack of support as more popular subtractive measures. In the absence of a known mechanism for combining implicit and explicit adaptation, either approach may be justified in some cases. Perhaps one of these subtractive measures is slightly better than the other depending on whether the study is primarily focused on explicit or implicit adaptation. However, neither should not be relied on indiscriminately, and a better alternative is needed.

Most current models of adaptation combine separate (neural) processes by adding their outputs. Here we chose the two-rate model (Smith et al., 2006) as an example since it is well known and yet still relatively straightforward. While the original study publishing the model convincingly shows there are multiple processes at play in adaptation, the mechanism used to combine the output of separate processes in this model is not necessarily neurally plausible. Crucially, this is not limited to this model but extends to any model using linear addition of neural processes. Neural net-based models would be an exception. Regardless, not only are the terms in our equations important, so are the operators. That is, while using addition is a good simplification while modeling, we should keep in mind that it is a simplification that does not necessarily reflect actual neural processes.

Some models explore new directions however, suggesting that separate adaptation processes can be subadditive (Albert et al., 2022), which could correspond to “loose” additivity here, depending on the fitted weights. Others use Bayesian mechanisms (Berniker and Kording, 2011; Tsay et al., 2022), such as MLEs, that are well-known in the literature on multimodal perception (Ernst and Banks, 2002). However, neither of these can explain the data we considered here.

In some experiments or measures this study relies on, the magnitude of either process could have been systematically misestimated. For example, some decay might occur between regular reach trials and whenever a measure of implicit adaptation is taken, resulting in underestimation. Or perhaps, between reaching with and without a strategy, the strategy is not fully disengaged resulting in overestimation of implicit adaptation. Alternatively, if participants only train with a single target, the largest reach aftereffects (or strategies) do not necessarily occur at the trained target, so that tests at the trained target would underestimate implicit (or explicit) adaptation. There might also be other reasons for systematic under- or overestimating of either implicit or explicit adaptation. And at first glance, the aggregate data (Fig. 7*A*) indicates a linear relationship with systematically underestimated implicit adaptation. We have tried to overcome this with our “loose” additivity approach. Here a weight is applied to both estimates of implicit and explicit adaptation before they are added as a prediction of total adaptation. While “loose” additivity fared somewhat better in some cases (Figs. 5, 6), it does not seem that systematic underestimation (or overestimation) for any reason can explain why strict additivity does not hold up in the current study.

The reanalysis of aggregate data from across the field shows a weak relationship between measures of implicit and explicit adaptation. And while we did not see any relationship in our own data set, it seems more plausible that the two interact in overall behavior. What, then, do we know about how implicit and explicit processes combine? First, given the lack of clear patterns, either the measures (Maresch et al., 2021b) or the underlying implicit and explicit adaptation processes could be highly variable and may saturate (Kim et al., 2018)—or both. Second, the mechanism by which the two processes combine is unknown, but not strictly additive. Third, we should consider that this relationship is likely influenced by other factors such as interactions between processes (Albert et al., 2022; Tsay et al., 2022), task contexts (Bond and Taylor, 2015; Modchalingam et al., 2019; Gastrock et al., 2020; Decarie and Cressman, 2022), or goals or motivations of participants. Beyond motor adaptation, these notions may apply to other types of learning as well.

What we learn from this study can be summed up in two recommendations. First, in order to investigate implicit and explicit processes, we should use independent measures of both. Currently, it seems that the best candidates are reaiming responses for explicit, and strategy exclusion trials for implicit, adaptation, although it is also unknown how neural adaptation processes map onto these behavioral measures. Second, the field should try to understand the (neural) mechanism by which various adaptation processes are combined to shape behavior.

## Synthesis

Reviewing Editor: David Franklin, Technische Universitat Munchen Decisions are customarily a result of the Reviewing Editor and the peer reviewers coming together and discussing their recommendations until a consensus is reached. When revisions are invited, a fact-based synthesis statement explaining their decision and outlining what is needed to prepare a revision will be listed below. The following reviewer(s) agreed to reveal their identity: Jean-Jacques Orban de Xivry.

Your manuscript has been reviewed by two experts in the field. Although both are in general positive about your manuscript and agree that this is an important topic and contribution to the field, we all think that there are still some areas of concern that need to be addressed. In particular, we would like to highlight four key concerns that we share after we came together to discuss the paper. First, the literature review of previous attempts could be strengthened. It seems to suggest that no one has tested whether there is additivity, whereas there has been some work on this previously using correlations - just not as extensively as you do within this paper. You can and should be critical of previous work but some discussion of the previous attempts to do this would help balance the manuscript. Second, we are concerned about the PDP probes - as the instructed group is a clear outlier - and wonder whether the subjects understood the instructions. Third we have concerns about the fitting of the state-space model with the data and would suggest to remove it as it adds little to the manuscript and is unlikely to be well constrained by the experimental design. Finally, we think you need to discuss plan-based generalization and temporal decay as potential reasons for the lack of correlation. We particularly highlight the second and fourth concerns. I include both independent reviews below to see the specific concerns and suggestions of the reviewers prior to our discussion. I encourage you to revise the manuscript with these key concerns in mind.

Reviewer 1:

Apologies if I wasn't clear in my (admittedly) long-winded comments in my previous review because many of the issues I raised still persist in the revised manuscript. Below I pasted my original comment, along with author's response, and my current response below.

I previously wrote: "To my knowledge, the most studies attempting to estimate implicit adaptation, when a form of aiming reports are used (i.e., subtraction method), also measure aftereffects in a no-cursor block where participants are instructed to refrain from aiming. In effect, this is the same as the exclusion trials from the process dissociation procedure. I think the authors acknowledge this in their meta analysis (Table 1), but the phrasing on page 2, line 35, makes it sound as though the subtraction method has not been verified yet accepted as a valid measure. In the original report of this method, Taylor et al. 2014, did perform a correlation analysis between the subtraction implicit and aftereffect implicit and only found a weak correlation when averaged over several trials. When the analysis was restricted to just the first and last trial between the aftereffect and subtraction implicit the correlation was robust. In the present paper, it is clear that there is significant decay during the exclusion/inclusion blocks as evidenced by the time courses in the top-up blocks in Figure 2."

In the manuscript, the authors repeatedly claim that prior work has not tested the additivity assumption and there has been a systemic failure to measure implicit adaptation through independent methods. Furthermore, I feel like they stress this point with a captious tone throughout the manuscript, at least up until the discussion where they are a bit more magnanimous. As I pointed out in my previous review, the original study of the subtraction method (Taylor et al., 2014) did perform a correlation analysis. Without discussing this in the introduction, it could give the false impression to the reader that this has been completely overlooked by the field.

The authors do acknowledge this in the response to reviewers: "Indeed the average of these subtraction method has been shown in perhaps a handful of studies to map onto the average of the onset of reach aftereffects. However, to us that does not constitute exhaustive proof, nor does I necessarily extend to individual participants, and this seems not to be based on any plausible neural mechanisms."

I agree with the authors that comparing the final state of implicit adaptation through the subtraction method with the onset of reach aftereffects is not exhaustive proof. Performing the individual differences correlation or slope analyses with independent measures, as the authors have done in the current manuscript, would be better. However, I think this difference between comparing just the average and performing individual differences is important to discuss in introduction/framing of the manuscript. Otherwise, it would again give the false impression that prior work has been completely neglectful.

The authors also state in their response to reviews: "Nevertheless we have seen a number of studies that no longer bother to test if this relationship holds in their data while still using it to produce a pseudo-measure of implicit adaptation. In some cases the conclusions are then circularly given by the method. For example, if you want to conclude that implicit adaptation picks up the slack from explicit adaptation, you should not rely on implicit adaptation as the difference between total and explicit adaptation, but rather make sure that your measures are independent and as correct as possible.

Again, it is my impression that most studies using the subtraction method have included a washout block where subjects are instructed to stop using a strategy and receive no-feedback, which is similar to the PDP exclusion test, and, as such, provides an independent measure of implicit adaptation. However, they may have only compared the average of implicit via subtraction with the aftereffect. Below are a few places where the claim "you should not rely on implicit adaptation as the difference between total and explicit adaptation, but rather make sure that your measures are independent and as correct as possible" may not be true in the studies the authors cited.

Lines 18-20: "Some research relies on the notion that implicit and explicit adaptation are related, assuming they linearly add to total adaptation (Bond and Taylor, 2015; Sulzenbruck and Heuer 2009). Here, we test whether or not this additivity assumption holds"

Perhaps I'm misinterpreting these sentences, but my interpretation is that the cited articles did nothing to investigate or even test if implicit and explicit added up.

Bond and Taylor (2015) compared implicit adaptation measured via subtraction and aftereffects in the no-strategy, no-feedback block in all of the experiments. While they observed differences on average between them, they were not significant. The only reason to compare these two is to verify the assumption.

The whole purpose of Sulzenbuck and Heuer (2009) was to verify the additivity assumption. They varied knowledge of the gain perturbation between groups and compared the degree of learning with aftereffects, although not with subtraction method nor no-feedback/no-strategy blocks. They even discuss at length the assumption and perform correlation analyses to test the degree of additivity and independence of the processes. I think it would be unfair to these authors to claim that they were tacitly assuming they linearly added when they were in fact testing whether they actually added together to form total adaptation.

Line 44-47: "This indirect, 'quasi measurement' of implicit adaptation is then also used to draw conclusions about implicit processes of adaptation (McDougle et al., 2015; Miyamoto et al., 2020; Wilterson and Taylor 2021). Of course, if this is a valid approach, i.e., if the additivity assumption holds, there really is no need to measure which would make visuomotor adaptation experiments easier or shorter"

Again, I interpret these sentences as suggesting that these cited articles based all of their conclusions about implicit adaptation from subtractive measures and did not consider the relationship between implicit and explicit.

McDougle and colleagues (2015) included a no-strategy washout block, although it had clamped visual feedback, and a two-rate state space model that was required to fit both implicit via subtraction and "aftereffects". While they do directly test the additivity assumption, they report differences between implicit measured via subtraction and the clamped aftereffects (page 9577-9578 of that manuscript) and discuss several reasons why this might be. Nonetheless, they did include an independent test of implicit adaptation, which isn't unlike the PDP exclusion procedure.

While Miyamoto and colleagues (2020) did not include an independent test of implicit adaptation, they designed their perturbation to separate out explicit and implicit by spectral analysis. Furthermore, they specifically tested their relationship performing perturbation-driven and perturbation-free correlations and residuals from the additivity model. It would be hard to conclude that this study was not testing the relationship between explicit and implicit adaptation processes.

Wilterson and Taylor (2021) used two methods to make claims about implicit adaptation, the subtraction method and no-feedback/no-strategy washout. They examined the correlation between the two measures, and found no correlation between, as in Maresch and colleagues (2021) and the current manuscript. While there isn't a correlation, the authors used both measures to reach their conclusion (that implicit adaptation was limited).

I realize that I'm being pedantic here, but I think these nuances and details are important and should be addressed in the manuscript. If not, then a reader, new to the field, could come away with the impression that testing the relationship between explicit and implicit hasn't been much of a focus in the field. Likewise, they could get the impression that methodological differences haven't been considered.

Previously I wrote: "we know that implicit adaptation decays quite rapidly and has been suggested to have temporal and labile components (Hadjiosif and Smith 2013; Joiner et al., 2017)....In the present paper, it is clear that there is significant decay during the exclusion/inclusion blocks as evidenced by the time courses in the top-up blocks in Figure 2. I think the authors should consider the effect of temporal decay, possibly examining how their correlations change with increasing the trials in the inclusion/exclusion blocks (they could use false discovery rate to handle all the multiple comparisons). This would help ensure that decay couldn't be the primary reason for the lack of correlation between explicit and implicit measures."

In the response to reviewers, the authors stated: "In our own experiment, we fail to observe any decay in our measures. The exclusion trials from the aiming group were already shown (now in Fig 4D). Although split into 2 subgroups there, neither of those appear to decay as well. We've added a figure here to illustrate the group average (after removing individual baseline biases) across all 6 blocks of no- cursor trials. As is clear, there is no decay, so that we can't estimate the decay to account for it. Since a larger number of data points will result in a more robust estimate of the mean, we have left those data as they were.

We've also added a new incomplete data set from our own lab ("D'Amario et al., unpublished") for other reasons (see below). In this data set there can not be any decay, since each measurement is a single trial, but there are many of them."

There still lacks sufficient description of the state-space model and Figure 4D to really understand what you did here, which I raised in my previous review where I asked for more description in both the text and figure. I think the authors should provide the fits of the state-space model to the hand angles. Without them, how do we know if the model fits are even in the ballpark of the data? Furthermore, are you fitting during the PDP blocks where no cursor feedback is provided? These no cursor trials would be important for the model to estimate a good forgetting factor over the whole experiment.

Putting this aside, I'm assuming that 4D is showing exclusion trials (implicit adaptation). If that is the case, then it does not appear that there is much decay during the PDP exclusion procedure. The figures included in the response to review, which show the no cursor trials over all 6 blocks also appear to show no decay in either exclusion or inclusion trials. So if there is no decay, then what explains the clear learning curves when the rotation is turned back on during the top-up trials (Figure 2A)?

In my previous review, I wrote "The behavior for the control and aiming groups in the inclusion block are puzzling. The behavior in the inclusion block should be the same as the adaptation block, however, they appear to be radically different (Fig. 2B). The only differences are participants are provided an instruction "For THESE trials, make full use of any strategies you learned just now" and there's no cursor feedback. So either the instructions have changed their behavior or the no cursor feedback changed the behavior. Either way, the PDP procedure on inclusion trials may be problematic. I would suggest performing a correlation between the end of the adaptation block and the inclusion trials. If my logic or understanding of their methods isn't flawed, then in theory they should have a strong correlation but based on the data (Fig. 2B) I don't suspect they will."

Perhaps my suggestion of a correlation analysis wasn't clear or ended up being a red herring. My concern is the dramatic differences during the include trials between the control, instructed, and aiming groups (as shown in Figure 2B). For the include trials, why does only the instructed group show complete learning (~30 degrees) while the control and aiming groups show only half learning (~15 degrees)? Why would there be any difference between these groups given that all groups show equivalent performance in the adaptation block and exclude trials?

I suspect the authors will respond that these inconsistencies underscore their claim that the measures (and implicit/explicit in general) do not add up. Couldn't an alternative explanation be that the PDP procedure doesn't work as intended and/or subjects didn't understand the instructions? I feel the authors have to discuss why the instructed group shows such a dramatic difference from the other groups.

In my previous review I wrote: "The authors should also consider the influence of plan-based (or aim-based) generalization (see Day et al., 2016; McDougle et al., 2017; Schween et al., 2018). These studies have found that the generalization function of implicit adaptation appears to peak around the intended aim location. When participants are instructed to not use any aiming strategy on exclusion trials, and there is still some degree of aiming at the end of the training block, then they would only be assessing the tails of an implicit generalization function, which could also have complex interactions with generalization functions of a neighboring target (implicit generalization width is on the order of a standard deviation of 20-30). Without having a densely sampled workspace during the exclusion block, this cannot be ruled out. Furthermore, Braynov et al., 2012 and Pearson et al. 2010 have reported gain-field like composite generalization functions - the generalization function can have a flat offset in the tails. As a result, this could not only affect their estimate of implicit adaptation on exclusion trials in their behavioral study but also the data used in the meta analysis may also be subject to this complexity."

The authors wrote in the response to reviewers: "In the data sent to us by Dr. Raphael Schween, there was a generalization effect for the estimates of implicit adaptation, and we controlled for that effect (as mentioned in Table 1).

In table 1 it says that generalization was accounted for in Schween et al studies by "average across targets straddling the averaged generalization curve's peak." Does this mean that you only examined implicit adaptation at a few target locations that coincided with the generalization curve's peak? How do you account for which trial number it was during the washout block, given that there's typically temporal decay? Or is there no decay in this study either?

The authors continued: "In all other data sets, training was done with multiple targets spanning at least 90 degrees, and testing was done at the same targets, such that generalization is not an issue. That is, we have already corrected for generalization where necessary."

Having targets span at least 90 degrees doesn't address the issue of generalization. It is the breadth of training that matters, but the spacing between the targets. The width of implicit adaptation's generalization function is on the order of 20-30 degrees, so you can expect that the degree of implicit generalization is 50% less only ~25 degrees away. So unless you considered only studies with more than 16 targets, then you can expect dips in implicit in between the targets. Furthermore, if there is plan-based generalization, then you would have to be looking at where they aimed and not the actual visually-displayed target. Finally, it has been reported that 95% of temporally-volatile adaptation decays in 15-25 seconds (Sing, Najafi et al., 2009; Hadjiosif, Petreska, and Smith 2012; Zhou, Fitzgerald et al., 2017; Hadjiosif, Morehead, and Smith 2023), so you would need to account for the angular separation (or time) between the last time they visited a target with feedback and the first time they reached to that same target without feedback.

The impact (and interaction) of plan-based generalization and temporal decay could be an interesting discussion point in the paper. Given that most studies use multiple targets these two factors would be in play. It could account for the difference in implicit adaptation observed with the subtraction method during rotation training and implicit adaptation observed during no-feedback/no-strategy washout blocks (or PDP exclusion trials). The contribution of temporally-volatile implicit adaptation likely decays by the time a PDP procedure begins.

In sum, I still think there are a number of points that the authors should consider. My hope is that my quibbles here could result in a more in-depth narrative and discussion in the manuscript and, hopefully, elevate the potential impact their findings could have on the field.

Reviewer 2:

I read with interest this paper that demonstrates that total adaptation is not simply the sum of its explicit and implicit components. I believe that the authors have done a good job in answering most of the previous comments (those were not my comments, I was not a reviewer for the first round).

The authors should further improve their response with respect to plan-based generalization (Day paper or McDougle one). Plan-based generalization means that, maybe, total adaptation is equal to the sum of the explicit and implicit components if the participants aim at the aiming point (where implicit adaptation is maximum and not if they are aiming at the target (as his done in exclusion trials). In short, in exclusion trials, one measures I(E), which is dependent on how big the explicit component is. I(E)=g(E) * I where g(E) is a generalization function around the aiming direction and I is the actual implicit component of adaptation. This means that I(E) is always an underestimation of the actual implicit component of adaptation. How does that affects the additivity hypothesis and the conclusion of the current paper?

I would like the authors to also discuss and maybe even investigate the validity of the PDP.

1. Does total adaptation correlates with inclusion results? If not, how should we interpret the inclusion trials?

2. What is the evidence that the inclusion trials are actually meaningful and that the participants are not completely unsure about what they should do? This is by far the most variable condition (compared to adaptation and exclude). Isn't this a sign that the inclusion condition is problematic?

3. For Fig. 2, how does total adaptation - exclude correlate with both aims and implicit measures? Are aims negatively correlated with implicit measure (explicit) ?

Now, I think that is important for the paper to have a critical view on the PDP but not all evidence rests on this technique.

Finally, I am unconvinced that the part of the state-space-model is interesting. Fitting the two-state model on normal learning curve is ill-defined because there is not enough variation of the deviation outcomes to provide reliable estimate of the different parameters. The cross-validated estimation of parameters would probably yield very large confidence intervals. I would remove that section for the manuscript as it distracts the reader from the main point.
